# Supplementary material for: Causal Relationship between Aging and Anorexia Nervosa: A White-Matter-Microstructure-Mediated Mendelian Randomization Analysis
Source: Biomedicines. 2024 Aug 16;12(8):1874. doi: 10.3390/biomedicines12081874 (PMC11351342; doi:10.3390/biomedicines12081874)
Supplement: Supplementary file 1 [file biomedicines-12-01874-s001.zip › Supplementary Figure/Figure S4/Singe SNP plot of MR analysis for TL to ubm-b-1467.pdf]

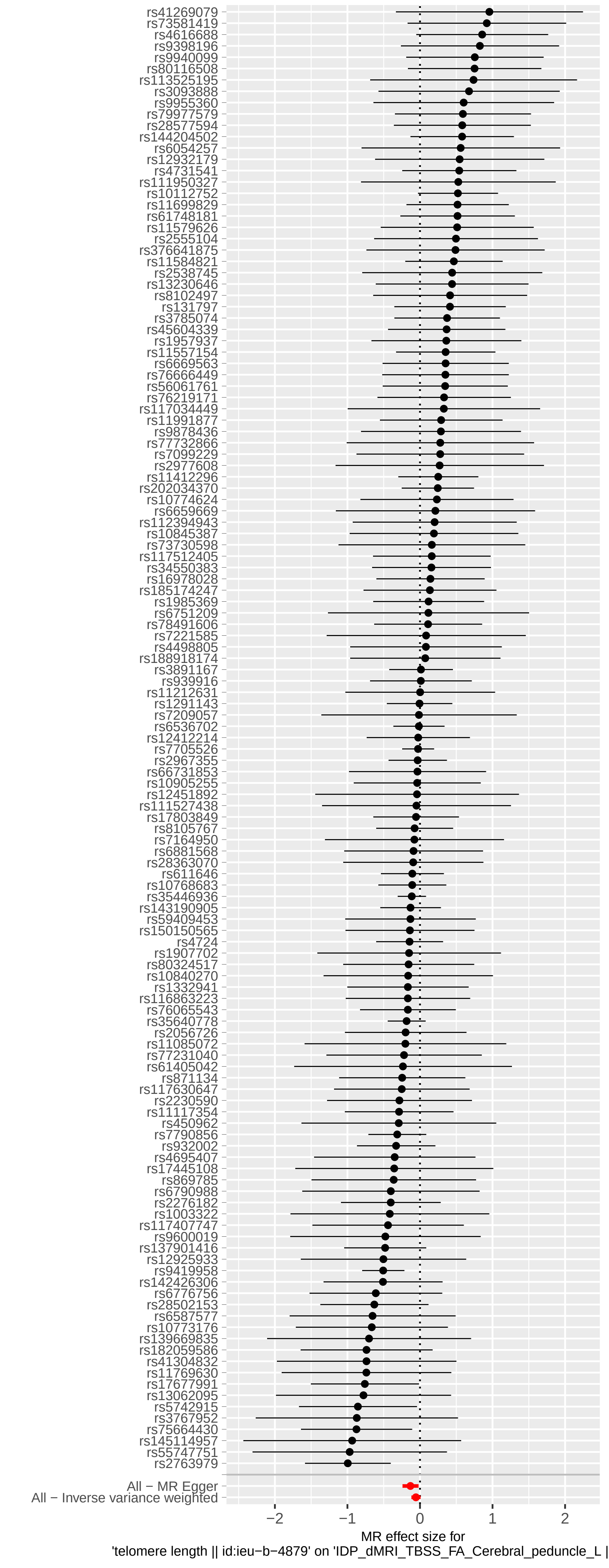

rs41269079
rs73581419
rs4616688
rs9398196
rs9940099
rs80116508
rs113525195
rs3093888
rs9955360
rs79977579
rs28577594
rs144204502
rs6054257
rs12932179
rs4731541
rs111950327
rs10112752
rs11699829
rs61748181
rs11579626
rs2555104
rs376641875
rs11584821
rs2538745
rs13230646
rs8102497
rs131797
rs3785074
rs45604339
rs1957937
rs11557154
rs6669563
rs76666449
rs56061761
rs76219171
rs117034449
rs11991877
rs9878436
rs77732866
rs7099229
rs2977608
rs11412296
rs202034370
rs10774624
rs6659669
rs112394943
rs10845387
rs73730598
rs117512405
rs34550383
rs16978028
rs185174247
rs1985369
rs6751209
rs78491606
rs7221585
rs4498805
rs188918174
rs3891167
rs939916
rs11212631
rs1291143
rs7209057
rs6536702
rs12412214
rs7705526
rs2967355
rs66731853
rs10905255
rs12451892
rs111527438
rs17803849
rs8105767
rs7164950
rs6881568
rs28363070
rs611646
rs10768683
rs35446936
rs143190905
rs59409453
rs150150565
rs4724
rs1907702
rs80324517
rs10840270
rs1332941
rs116863223
rs76065543
rs35640778
rs2056726
rs11085072
rs77231040
rs61405042
rs871134
rs117630647
rs2230590
rs11117354
rs450962
rs7790856
rs932002
rs4695407
rs17445108
rs869785
rs6790988
rs2276182
rs1003322
rs117407747
rs9600019
rs137901416
rs12925933
rs9419958
rs142426306
rs6776756
rs28502153
rs6587577
rs10773176
rs139669835
rs182059586
rs41304832
rs11769630
rs17677991
rs13062095
rs5742915
rs3767952
rs75664430
rs145114957
rs55747751
rs2763979
All − MR Egger
All − Inverse variance weighted
−2
−1
0
1
2
MR effect size for
'telomere length || id:ieu−b−4879' on 'IDP_dMRI_TBSS_FA_Cerebral_peduncle_L |
